# Influence of Academic Training and Professional Experience on the Management of Deep Caries Lesions

**DOI:** 10.3390/healthcare12191907

**Published:** 2024-09-24

**Authors:** Sebastiana Arroyo-Bote, David Ribas-Perez, Catalina Bennasar Verges, Diego Rodriguez Menacho, Paloma Villalva Hernandez-Franch, Ignacio Barbero Navarro, Antonio Castaño Séiquer

**Affiliations:** 1 University of the Balearic Islands, 07006 Palma de Mallorca, Spain; s.arroyo@eua.edu.es (S.A.-B.); c.bennasar@eua.edu.es (C.B.V.); 2 Department of Stomatology, University of Seville, 41001 Seville, Spain; drmenacho@us.es (D.R.M.); ibarbero2@us.es (I.B.N.); acastano@us.es (A.C.S.)

**Keywords:** caries lesions, pulp vitality, dentist behavior, academic training, conservative dentistry

## Abstract

Background/Objectives: Managing caries lesions that affect the inner third of the dentin is crucial to ensuring pulp vitality; the clinician must make decisions that will affect the vitality of the tooth. Our purpose is to understand the behavior of Spanish dentists in treating deep cavities and to examine whether variations exist based on their academic training and/or years of professional experience. Methods: This study was approved by the ethics committee of the Balearic Islands CEI-IB. A survey was conducted using the SurveyMonkey platform with 11 questions, the first 4 of which focused on defining the characteristics of the respondents. The following six concerned a clinical case of deep caries in tooth number 4.7, and the last regarded the opinion of the actual treatment of the case. The survey was sent by email in April 2022. The results were analyzed with the SPSS 29.0 program using the chi-square test. Results: A total of 347 responses were obtained (93.95%), and those surveyed stated that they apply minimal intervention concepts in their treatments, with 90.49% performing conservative dentistry treatments daily. A total of 56.48% of the respondents had bachelor’s degrees, 12.39% had graduated, 33.14% had a postgraduate degree, 38.90% had a master’s degree, and 17% had a doctorate. Most (40.63%) had been in professional practice for 16–30 years. Conclusions: Significant differences were identified regarding years of professional experience in terms of decision-making in methods of treatment and the choice of materials used for pulp protection. Likewise, significant differences were found regarding the academic training of the respondents, the cavity cleaning method selected, and the use of chemical substances for removing carious dentin. We can conclude that academic training and years of professional practice influence decision-making at some points in treating deep caries lesions.

## 1. Introduction

Managing caries and carious lesions represents a daily challenge for the clinician in making preventive and restorative decisions. Articles have been published to help clinicians evaluate the risk of caries and make decisions regarding a comprehensive treatment plan for the disease and restorative treatments for carious lesions [1,2,3,4].

Caries begins with the demineralization of the enamel and progresses inside the tooth until it reaches the dental pulp. The dentin–pulp complex activates the defensive processes of the tooth through internal tubular calcification and the formation of neodentin. The relationship between the aggressiveness of caries and the effectiveness of the tooth’s defenses will give rise to different clinical situations regarding the activity of cavities, becoming inactive in some situations.

Classically, the clinical attitude focused on the operative restoration of lesions, regardless of the degree of caries activity, even in lesions that affect only the enamel and external third of the dentin. These operational restoration criteria have varied based on minimal intervention dentistry and scientific evidence [5]. It is important to establish the level of involvement of the dental tissues due to caries and establish the patient’s risk of caries so the most appropriate and comprehensive treatment of the disease can be indicated, avoiding operative treatments that entail significant destruction of the dental tissues and offering the patient the possibility of protection and defense against the progression of caries. However, studies focused on determining the conservative attitude of minimal intervention or interventionist approaches, in relation to years of professional experience and training in cariology, show contradictory results [6]. In a study conducted in London with 217 general dentists, a more interventionist attitude was demonstrated in more experienced dentists; however, less-experienced dentists performed less conservative cavities, which can be explained from different points of view but which highlights the lack of judgment in the management of carious lesions [6].

Deep carious lesions in the inner third of the dentin, very close to the pulp, are at significant risk of exposing the pulp when the carious dentin is removed. In such scenarios, contemporary principles of minimal intervention advocate for the targeted removal of caries and the application of bioactive materials that facilitate the remineralization of demineralized dentin. This approach enables a highly conservative removal of damaged tissues, extracting only the disintegrated dentin while preserving the demineralized, thus preventing pulp exposure. This treatment contrasts sharply with traditional cleaning methods, which recommended completely removing all demineralized dentin until reaching hard dentin, thereby increasing the risk of pulp exposure. As we have previously stated, there is a lack of uniformity in the way of acting in these injuries.

On the other hand, new biologically active materials have been introduced in recent years and have been changing the modus operandi of dentists.

Mineral Trioxide Aggregate (MTA) was developed by Loma Linda University (Loma Linda, CA, USA) and was commercialized in 1998 (ProRoot MTA, Dentsply, York, PA, USA). It consists of tricalcium and dicalcium silicate particles, tetracalcium ferric aluminate, calcium sulfate dihydrate, tricalcium oxide, silicate oxide, and bismuth trioxide. In the setting process, MTA initially presents a pH of 10.2 that rises to 12.5 within the first hours [3]. 

MTA is indicated for endodontic treatment and pulp protection in treating teeth with pulp exposure by direct coating or for total or partial pulpotomy [4]. Biodentine is a calcium silicate-based biomaterial. Its composition includes tricalcium silicate, zirconium oxide, calcium oxide, calcium carbonate, iron oxide pigment in the powder, and calcium chloride and polycarboxylate in the aqueous solution. Biodentine is used in conservative dentistry treatments in pulp capping (direct and indirect), pulpotomy, endodontic drilling, and apexification, among others [4].

This study aims to analyze how Spanish dentists face this treatment, correlating their attitude to age and professional training.

## 2. Materials and Methods

This research was approved by the ethics committee of the Balearic Islands CEI-IB in a session held on March 30 (no. 04/2022), CEI Code: IB 4142/20 PI.

**Figure 1:**
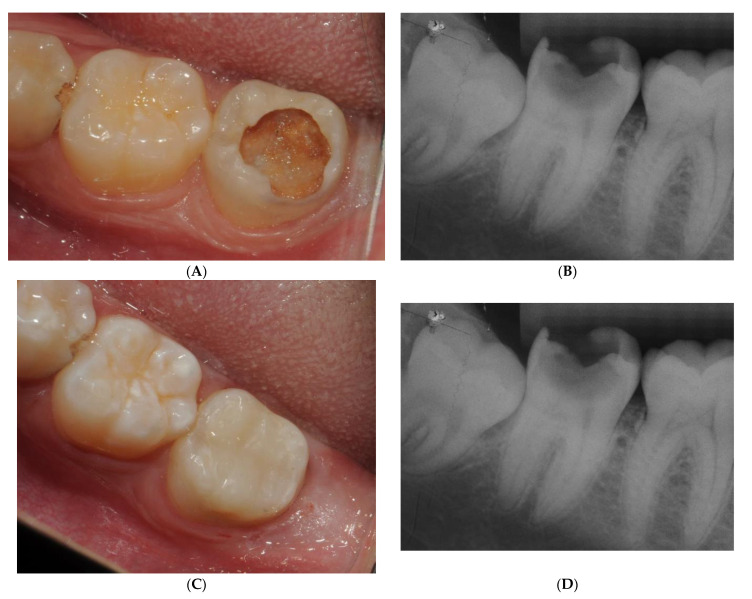
(**A**,**B**) Initial stage of deep caries in a second permanent molar. (**C**,**D**) Tooth treated with selective caries removal, bioactive material (Biodentine(r)) application, and filled with bulKfill.

A validated survey was conducted using the SurveyMonkey platform of 13 questions based on a clinical case (Figure 1A,B) of a caries lesion that had been treated with selective caries removal, application of bioactive material (Septodont, Saint-Maur-des-Fossés, France, Biodentine), and filled with bulKfill composite and universal adhesive with the selective enamel etch technique (Figure 1A,B). A group of professors from Seville University and ADEMA Faculty of Baleares designed and validated the survey, which underwent a preliminary pilot study.

The survey was divided into three parts to achieve the objectives. The first part aimed at knowing the respondent’s characteristics and consisted of 4 questions. The second part was based on the clinical case of a 25-year-old patient with an active, deep, and cavitated caries in 4.7 with sensitivity and food retention (Figure 1A). This had positive but slightly increased vitality tests. In the periapical radiograph, deep demineralization can be observed very close to the pulp cavity (Figure 1B). No widening of the periodontal ligament or periapical lesions was observed. Given this clinical situation, 7 questions were formulated focused on knowing the attitude of the dentists and responding to the secondary objectives. In the third part, the conservative clinical resolution and minimal intervention carried out in the clinical case were presented (Figure 1C,D); from here, 1 question was asked to gauge the respondents’ opinions.

The survey was emailed nationwide to the dentists in the “Odontólogos de hoy” magazine database in April 2022.

Surveys that were not completed or had any errors were excluded.

A descriptive statistical study was conducted, and the results were analyzed using the SPSS 29.0 program. The percentage values for frequencies and the chi-square test to assess the differences in prevalence were analyzed using a value of *p* < 0.05 as statistically significant.

## 3. Results

The survey was answered by 347 dentists; the results are shown in Table 1. A total of 93.95% of those surveyed stated that they apply minimal intervention concepts in their treatments, with 90.49% performing conservative dentistry treatments daily. Regarding academic training, 56.48% had a bachelor’s degrees, 12.39% had graduated, 33.14% had a postgraduate degree, 38.90% had a master’s degree, and 17% had a doctorate. Regarding years of professional practice, most (40.63%) were in professional practice for 16–30 years (Table 1).

Regarding the attitude towards the clinical case presented in question 5, 54.17% would opt for the preservation of the pulp and the definitive treatment of the case with a direct or indirect pulp capping and definitive filling. There were significant differences in terms of the years of professional practice of the respondents, with a *p* = 0.019; however, there were no significant differences in the professionals’ academic training. It is important to highlight that 14.70% would initially opt for root canal treatment as the first treatment option.

Regarding the methods and techniques of cavity removal, the majority (66.28%) would use a round burr with rotating instruments (blue contra-angle) and a manual excavator, with practically the same percentage using chemical methods to detect the carious lesion as those who would not, presenting significant differences in academic training (*p* = 0.008 and 0.048, respectively.) A total of 66.86% of those surveyed based their treatment on clinical symptoms, with the patient’s age, for 19.31%, being the criterion to determine the treatment, and only 12.97% basing their treatment on the radiographic image of the case. There are significant differences with respect to years of professional practice, but there are significant differences in academic training (*p* = 0.004) (Table 2).

The three most used materials in pulp protection are Biodentine 38.90%, calcium hydroxide 26.80%, and glass ionomer 21.04%, MTA 11.82%. There were significant differences in years of professional practice (*p* < 0.001) but not in academic training (*p* = 0.111).

The cavitation filling material selected by the majority was conventional standard composite, at 65.48%, followed by bulk composite fill, at 31.99%. There were no significant differences either in years of professional practice or academic training. Regarding the adhesive technique used, 61.67% would use universal adhesive with the selective etching technique, followed by 25.65% who would use one-component total etching adhesive, and only 10.37% would use self-etching adhesive without selective etching. There were no significant differences in years of professional practice, but there were significant differences in academic training (*p*= 0.033) (Table 3 and Table 4).

## 4. Discussion

Approaching the treatment of deep caries lesions is a challenge for clinicians. Before starting it, a full clinical study of the state of caries activity must be carried out, as well as a radiographic study and a thorough pulp diagnosis [7], since the preservation and vitality of the pulp must be the first objective of the restorative treatment.

In deep cavities, it is very easy to expose the pulp through cleaning methods; therefore, the first step is to determine the method and approach for cavity cleaning. Classically, the removal of all the carious dentin is carried out, which involves the elimination of the affected and infected dentin; in deep lesions, this can cause the exposure of the pulp and, therefore, the need to perform total removal treatments of the pulp on many occasions. To prevent this clinical scenario, and in accordance with a minimally invasive strategy supported by bioactive materials, the technique of selective caries removal has gained traction. This method involves retaining demineralized dentin to facilitate remineralization through the application of materials used as cavity bottoms, thereby avoiding the complete removal of carious dentin and subsequent pulp exposure, as recommended by the European Society of Endodontics [7]. This selective removal of caries can be carried out in a single session when the definitive filling is performed. In a recent study, Savolainen N et al. [8] concluded that selective caries removal in a single session is significantly more cost-effective in preventing root canal treatment and pulp exposure than gradual caries elimination. A recent meta-analysis by González-Gil D et al. [9] was unable to demonstrate significant differences between methods of selective caries removal and traditional methods. However, there is evidence of greater success in selective caries removal, exhibiting a lower percentage of pulp exposure. Another study by Fraser J. and MacInnes A. [10] reported that in the short term (1.5 years of treatment), higher success rates are found in teeth treated with selective caries removal than those treated with classical methods. However, the results are similar in the long term (5 years), with no differences found depending on the restorative material used.

Another study by Crespo-Gallardo I. et al. [11] concluded that total caries removal is the treatment of choice in teeth with deep lesions and reversible pulpitis, demonstrating that new ideas about caries lesions and the more conservative approach to deep caries lesions have not yet been incorporated by dentists in their usual clinical practice. It is important to highlight that training in cariology is necessary, since our study reveals that a significant percentage (14.70%) would directly enhance the total removal of the pulp, and 7.78% would not use any pulp protection material. While most dentists prioritize pulp protection and preservation, it is concerning that over 20% of those surveyed do not implement any treatment aimed at pulp maintenance in cases of deep carious lesions, underscoring the non-conservative approach adopted by a substantial proportion of dentists in our study. Similar results were obtained by Elkady and Khater [12] in a survey of Egyptian dentists, where 80.8% eliminated cavities based on the hardness and color of the dentin. In comparison, 67% eliminated caries until hard dentin remained, not applying concepts of minimal intervention. Similar results were obtained by Drachev SN et al. [13] in a study of Russian dentists. Our findings indicate that 93.95% of respondents claim to implement minimal intervention concepts; however, 14.70% would undertake root canal treatment for deep carious lesions with reversible pulpitis, suggesting a discrepancy between the principles of minimal intervention dentistry and clinical practice. These results align with the systematic review and meta-analysis by de Moura RC et al. [14] on dentists’ knowledge, attitudes, and practices in minimal-intervention dentistry. Moreover, Al-Asmar AA et al. [15], in a large survey of Jordanian dentists, concluded that in operative dentistry, evidence-based research is not implemented clinically. The dental curriculum must be constantly updated and modified to optimize the relationship between evidence-based dentistry and clinical decision-making. However, this discrepancy is not only detected in the management of deep caries but also in the management and attitudes towards incipient caries lesions or the management of patients at risk of caries [16,17].

Another interesting point is the use of chemical substances that help eliminate carious lesions. One of the most used is Papain, the effects of which on dentin and pulp cells are not completely known. Bastos, LA. et al. [18] demonstrated that papain-based gel presents concentration-dependent cytotoxicity, without affecting cell viability, for dental pulp cells and macrophages. Interestingly, the gel inhibited the differentiation of pulp cells but modulated the activation of macrophages stimulated with LPS, suggesting that in the dental pulp tissue, Papacárie Duo^®^ would impair reparative dentinogenesis but could activate macrophages to play their role in defense and inflammation. In a study examining three techniques for the removal of carious dentin, Al-Sagheer RM et al. [19] determined that the chemical method using Papain (Brix 3000) results in a residual cavity dentin surface characterized by a higher organic component and reduced phosphate content, alongside more permeable tubules, compared to rotary methods. The researchers suggest that this approach offers a promising strategy for clinical dental practice. However, in our research, only 14.20% of respondents would use chemical methods and manual removal with an excavator to clean and disinfect the carious lesions, although when asked about question 7 of whether they would “use chemical methods to detect the carious lesion and help the selective removal of caries”, 48.13% answered yes, which again demonstrates that there is a dissociation between what they would probably do and what is actually performed, since 66.28% chose the removal of carious dentin with a contra-angle rotary instrument with round bur for dentin removal with cooling and a manual excavator.

The answer to question 8 is interesting, as it refers to what decision-making is based on when treating vital deep caries lesions. Most lend greater importance to the patient’s clinical picture than to the age of the tooth when there is scientific evidence proving that the patient’s age is fundamental to the success of direct and indirect pulp protection treatments.

Regarding the material that should be used for direct pulp exposures, the European Society of Endodontics recommends calcium hydroxide, and for indirect coatings, calcium hydroxide or glass ionomer [7]. In our research, the most used material is Biodentine for 38.90% of respondents, followed by calcium hydroxide at 26.80% and glass ionomer at 21.04%. Only 11.82% would use MTA, which contrasts with the study by Croft K et al. [20] on dentists in Finland, where the MTA obtained significant differences with the rest of the materials, especially in dentists who had attended training courses. However, another study in Lithuania [21] yielded similar results to ours, with 68% of dentists surveyed in 2021 using calcium silicate hydraulic cements. The recent work by Kunert M. et al. [22] on the mineralization capacity of different bioactive materials concluded that those best at inducing the remineralization of dentin are the classic calcium silicate cements, achieving better results than the materials modern resin-based bioactive. Pires PM et al. [23] reached similar conclusions, indicating that all ion-releasing materials examined in their study (glass ionomer cement, calcium silicone cement, and resin-modified calcium silicone cement) demonstrated mineral precipitation in demineralized dentin. However, calcium silicate-based materials specifically facilitated apatite precipitation and the gradual restoration of hardness in artificial carious dentin lesions. Furthermore, a review on the application of bioactive materials in coronal dentin [24] concluded that while most materials exhibit potential for bioactivity, there is a paucity of clinical studies that provide conclusive scientific evidence.

In our study, we observed that years of professional practice or academic training significantly influence some of the decision-making regarding the clinical case surveyed, coinciding with the results of Vianna, RF et al. [25], who concluded that the clinical decision-making by Brazilian dentists varies according to the professional profile, mainly in relation to the replacement of restorations for aesthetic reasons.

Recent studies aimed at studying the clinical behavior of universal dental adhesives used with different clinical strategies have determined that there is greater permanence of the restorations when they are used with selective enamel etching; however, they concluded that the clinical performance of the adhesive is good regardless of the clinical application strategy [26,27]. However, other recent research has concluded that the retention rate of fillings is lower when the adhesive is applied with the self-etching strategy. Maciel Pires P et al. [28] concluded that universal adhesives should be used without etching at the dentin level because they can compromise the quality of the resin–dentin interface.

Other studies aimed at determining postoperative sensitivity in restorations in the posterior sector (class I, II, and V) have found that the use of total-etching or self-etching adhesives does not affect the presence or degree of postoperative sensitivity, finding better performance regarding the aesthetic and marginal maintenance of the restoration in the use of adhesives with total etching [29]. Nonetheless, the clinical application strategy of universal adhesives cannot solely account for their adhesive properties, which also rely on the adhesive’s composition, as concluded by Hurtado et al. [30], and on the implementation of pretreatments, such as dimethyl sulfoxide, to enhance adhesion and prevent degradation over time [31].

The most frequently used direct restorative material in cavity restorations is composites. In our study, 56.48% chose conventional standard composite, 31.99% selected bulk-fill composite, and only 10.09% chose a compomer. Despite being the definitive restorative material most used in direct restorations, composites have some properties that make them controversial. Efforts are currently underway to chemically implement these materials to provide them with antibacterial properties and improve their biocompatibility [32,33]. The results of the recent research by Zhang, S et al. [32] are promising; the authors explain the development of with a three-dimensional structure and bioactive components of calcium, phosphorus, and fluorine and used versatile FUHA particles with different charge fractions as functional fillers to manufacture methacrylate-based composites. The results showed that FUHA with a loading of 50% by weight in the resin matrix provided the composite with excellent physicochemical properties, providing properties of dentin remineralization, cell viability, promotion of the mineralization of the stem cells of the dental pulp, and antibacterial properties. In addition, it presented good aesthetic and clinical handling characteristics. Likewise, work is being undertaken to develop bulk-fill composites free of BisGMA. [34].

## 5. Conclusions

After analyzing the results, we can conclude that academic training significantly influences decision-making concerning the treatment approach and the liner used for pulp protection. Likewise, years of professional practice significantly affect the method of cavity cleaning, the use of chemical substances to remove carious dentin, and the most important criterion in the decision-making process for the treatment of deep caries in vital teeth. No significant differences were identified in the years of respondents’ professional experience or educational qualifications about the adhesive technique employed or the restorative material chosen; most respondents continue to utilize traditional methods for removing carious dentin.

## Figures and Tables

**Table 1:** Responses on prior knowledge and educational data of the sample.

Questions	Answers	%
Do you think you apply the concepts of minimal intervention in your treatments?	Yes	93.95
	No	3.17
	Do not know, no answer	2.88
How many years have you been practicing professionally?	<5	9.51
	5–15	23.05
	16–30	40.63
	>30	26.80
Do you perform conservative dentistry in your daily practice?	Yes	90.49
	No	4.03
	Only sometimes	5.48
What university degree do you have? Mark the answers that you consider	1 Degree	12.39
	2. Bachelor’s degree	56.48
	3. Postgraduate	33.14
	4. Master	38.90
	5. PhD	17.00

**Table 2:** Percentage of responses to the questionnaire on the clinical case.

Questions	Answers	%
A 25-year-old patient comes to the clinic because he notices sensitivity and food retention on the right side of the jaw. On examination, the patient presented a cavitated caries lesion at 4.7, with slightly increased positive vitality tests. Deep demineralization is observed very close to the pulp cavity in the periapical radiograph. No widening of the periodontal ligament or periapical lesions is observed. Given this clinical situation, which of the following procedures do you think would be indicated?	1. Definitive filling without specific pulp protection	7.78
	2. Definitive filling with specific pulp protection (indirect or direct pulp capping)	54.17
	3. Temporary filling with specific pulp protection (indirect or direct pulp capping)	23.34
	4. Pulp treatment and definitive obturation	14.70
What methods and techniques would you use to clean and disinfect cavities?	1. Contra-angle rotary material with round bur for dentin removal with cooling	13.26
	2. Manual removal using an excavator	5.76
	3. Manual removal with chemical methods and excavator	14.12
	4. Contra-angle rotary material with round bur for dentin removal with cooling and manual excavator	66.28
	5. Does not know/Does not answer	0.58
Would you use chemical methods to detect carious lesions and help selectively eliminate caries?	Yes	48.13
	No	48.70
	Do not know, no answer	3.17
In deep vital teeth, the treatment that would be indicated is based, above all, on:	1. Age of the patient	19.31
	2. Clinical symptoms	66.86
	3. Clinical image or X-ray of the cavity	12.97
	4. Does not know/Does not answer	0.86
If you decide to do a filling with pulp protection, what would you choose first?	1. Glass ionomer	21.04
	2. Biodentine	38.90
	3. MTA	11.82
	4. Calcium hydroxide	26.80
	5. Does not know/Does not answer	1.44
What type of would you choose for filling the cavity?	1. Conventional standard composite	56.48
	2. Composite bulk fill	31.99
	3. Compomer	10.09
	4. Does not know/Does not answer	1.44
What adhesive technique would you use?	1. Single-component total-etch adhesive	25.65
	2. Self-etching adhesive with selective engraving	61.67
	3. Self-etching adhesive	10.37
	4. Does not know/Does not answer	2.31
The clinical case presented was resolved conservatively based, above all, on the patient’s youth and the possibilities of reparative dentin calcification. The cleaning of the caries was carried out using an excavator and chemical methods. Biodentine was used as an indirect pulp protector. The filling was performed with self-etching adhesive with selective etching and bulk fill composite. Does your choice of treatment coincide with the treatment given to the patient? Check more than one option if you consider it	1. Yes	31.70
	2. Only the cavity cleaning method	21.33
	3. Only in the pulp protector	23.34
	4. Only in the adhesive technique	41.21
	5. Only in the filling material	17.58
	6. No, I do not believe in conservative treatments for this type of cavities without performing root canal treatment.	8.65
In the clinical image, you can see the resolution of the case, and in the x-ray, you can see the evolution one year after treatment. What do you think?	1. Conservative methods with the application of bioactive liners are a real treatment option for deep cavities, so I agree with the treatment carried out.	89.63
	2. I do not believe in pulp protection, so I would perform root canal treatment	0.58
	3. Despite the scientific evidence of bioactive liners in pulp protections, I continue to perform treatments that assure me that the patient will not present complications, which is why I disagree with the treatment carried out.	6.05
	4. Does not know/Does not answer	3.75

**Table 3:** Responses according to years of practice. * *p* value < 0.05.

Questions	Answers	<5 Years	5–15 Years	16–30 Years	>30 Years	
		%	%	%	%	*p*
Question 5	1. Definitive filling without specific pulp protection	0.0	11.3	5.7	10.8	0.019 *
	2. Definitive filling with specific pulp protection (indirect or direct pulp capping)	60.6	62.5	55.3	43.0	
	3. Temporary filling with specific pulp protection (indirect or direct pulp capping)	27.3	16.3	20.6	32.3	
	4. Endodontic treatment and definitive obturation	12.1	10.0	18.4	14.0	
Question 6	1. Contra-angle rotary material with round bur for dentin removal with cooling	9.1	15.0	14.9	10.8	0.108
	2. Manual removal using an excavator	0.0	3.8	4.3	11.8	
	3. Manual removal with chemical methods and excavator	6.1	11.3	17.0	15.1	
	4. Contra-angle rotary material with round bur for dentin removal with cooling and manual excavator	84.8	70.0	63.1	61.3	
	5. Does not know/Does not answer	0.0	0.0	0.7	1.1	
Question 7	Yes	39.4	47.5	49.6	49.4	0.187
	No	54.5	52.5	45.4	48.4	
	Do not know, no answer	6.1	0.0	5.0	2.2	
Question 8	1. Age of the patient	9.1	16.3	21.3	22.6	0.142
	2. Clinical symptoms	60.6	68.8	69.5	63.4	
	3. Clinical image or X-ray of the cavity	27.3	13.8	9.2	12.9	
	4. Does not know/Does not answer	3.0	1.3	0.0	1.1	
Question 9	1. Glass ionomer	27.3	16.3	29.1	10.8	<0.001 *
	2. Biodentine	51.5	47.5	35.5	32.3	
	3. MTA	18.2	21.3	7.8	7.5	
	4. Calcium hydroxide	3.0	12.5	25.5	49.5	
	5. Does not know/Does not answer	0.0	2.5	2.1	0.0	
Question 10	1. Conventional standard composite	54.5	57.5	52.5	62.4	0.870
	2. Composite bulk fill	30.3	31.3	35.5	28.0	
	3. Compomer	12.1	8.8	11.3	8.6	
	4. Does not know/Does not answer	3.0	2.5	0.7	1.1	
Question 11	1. Single-component total-etch adhesive	15.2	23.8	27.7	28.0	0.177
	2. Self-etching adhesive with selective engraving	81.8	66.3	58.9	54.8	
	3. Self-etching adhesive	3.0	7.5	12.1	12.9	
	4. Does not know/Does not answer	0.0	2.5	1.4	4.3	

**Table 4:** Responses according to degree of study. * *p* value < 0.05.

Questions	Answers	Bachelor	Postgraduate	Master	Doctorate	
		%	%	%	%	*p*
Question 5	1. Definitive filling without specific pulp protection	8.1	11.0	6.0	6.8	0.889
	2. Definitive filling with specific pulp protection (indirect or direct pulp capping)	51.5	50.7	59.5	52.5	
	3. Temporary filling with specific pulp protection (indirect or direct pulp capping)	24.2	20.5	22.4	27.1	
	4. Endodontic treatment and definitive obturation	16.2	17.8	12.1	13.6	
Question 6	1. Contra-angle rotary material with round bur for dentin removal with cooling	14.1	17.8	12.1	8.5	0.008 *
	2. Manual removal using an excavator	8.1	2.7	5.2	6.8	
	3. Manual removal with chemical methods and excavator	7.1	5.5	19.8	25.4	
	4. Contra-angle rotary material with round bur for dentin removal with cooling and manual excavator	70.7	74.0	61.2	59.3	
	5. Does not know/Does not answer	0.0	0.0	1.7	0.0	
Question 7	Yes	38.4	49.3	56.9	45.8	0.048 *
	No	54.5	49.3	41.4	52.5	
	Do not know, no answer	7.1	1.4	1.7	1.7	
Question 8	1. Age of the patient	21.2	6.0	20.7	25.4	0.004 *
	2. Clinical symptoms	58.6	53.4	62.9	66.1	
	3. Clinical image or X-ray of the cavity	18.2	2.6	16.4	8.5	
	4. Does not know/Does not answer	2.0	0.9	0.0	0.0	
Question 9	1. Glass ionomer	23.2	20.5	20.7	18.6	0.111
	2. Biodentine	32.3	30.1	49.1	40.7	
	3. MTA	10.1	17.8	11.2	8.5	
	4. Calcium hydroxide	33.3	28.8	17.2	32.2	
	5. Does not know/Does not answer	1.0	2.7	1.7	0.0	
Question 10	1. Conventional standard composite	60.6	54.8	55.2	54.2	0.169
	2. Composite bulk fill	30.3	32.9	35.3	27.1	
	3. Compomer	6.1	12.3	7.8	18.6	
	4. Does not know/Does not answer	3.0	0.0	1.7	0.0	
Question 11	1. One-component total-etch adhesive	26.3	34.2	26.7	11.9	0.033 *
	2. Self-etching adhesive with selective engraving	55.6	53.4	66.4	72.9	
	3. Self-etching adhesive	15.2	11.0	6.0	10.2	
	4. Does not know/Does not answer	3.0	1.4	0.9	5.1	

## Data Availability

The data presented in this study are available on request from the corresponding author due to ethical restrictions.
